# Tunable *e*_g_ Orbital Occupancy in Heusler Compounds for Oxygen Evolution Reaction**

**DOI:** 10.1002/anie.202013610

**Published:** 2021-02-03

**Authors:** Mingquan Yu, Guowei Li, Chenguang Fu, Enke Liu, Kaustuv Manna, Eko Budiyanto, Qun Yang, Claudia Felser, Harun Tüysüz

**Affiliations:** ^1^ Max-Planck-Institut für Kohlenforschung Kaiser-Wilhelm-Platz 1 45470 Mülheim an der Ruhr Germany; ^2^ Max Planck Institute for Chemical Physics of Solids Nöthnitzer Straβe 40 01187 Dresden Germany; ^3^ Beijing National Laboratory for Condensed Matter Physics Institute of Physics Chinese Academy of Sciences Beijing 100190 P. R. China; ^4^ Department of Physics Indian Institute of Technology Delhi New Delhi 110016 India

**Keywords:** cobalt, heterogeneous catalysis, Heusler compounds, oxygen evolution reaction, water electrolysis

## Abstract

Heusler compounds have potential in electrocatalysis because of their mechanical robustness, metallic conductivity, and wide tunability in the electronic structure and element compositions. This study reports the first application of Co_2_ *YZ*‐type Heusler compounds as electrocatalysts for the oxygen evolution reaction (OER). A range of Co_2_ *YZ* crystals was synthesized through the arc‐melting method and the *e*_g_ orbital filling of Co was precisely regulated by varying *Y* and *Z* sites of the compound. A correlation between the *e*_g_ orbital filling of reactive Co sites and OER activity was found for Co_2_Mn*Z* compounds (*Z*=Ti, Al, V, and Ga), whereby higher catalytic current was achieved for *e*_g_ orbital filling approaching unity. A similar trend of *e*_g_ orbital filling on the reactivity of cobalt sites was also observed for other Heusler compounds (Co_2_V*Z*, *Z*=Sn and Ga). This work demonstrates proof of concept in the application of Heusler compounds as a new class of OER electrocatalysts, and the influence of the manipulation of the spin orbitals on their catalytic performance.

## Graphical abstract

Heusler compounds (Co_2_ *YZ*) with mechanical robustness, metallic conductivity, and wide tunability in the electronic structure and element compositions, are studied as a new class of oxygen evolution electrocatalysts. The *e*_g_ orbital filling of active Co sites was precisely regulated by varying the *Y* and *Z* sites of the Co_2_ *YZ* compounds, where the higher catalytic current was achieved for *e*_g_ orbital filling approaching unity.

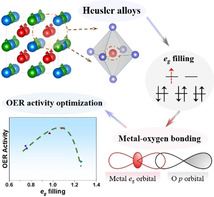

Heusler compounds are a large class of intermetallic compounds with the chemical formula *X*_2_ *YZ* or *XYZ* (also known as “half‐Heusler”), where *X* and *Y* are transition metals, *Z* is usually the main group element or a transition metal.^[1]^ Owing to the wide tunability in the electronic structure and constituent elements, Heusler compounds have shown plentiful magnetic and electronic functionalities, such as half‐metallicity, thermoelectric, superconducting, magneto‐caloric, magneto‐optical, spin transfer torque characteristics, as well as, topological insulator, magnetic Weyl fermion and magnetic Skyrmion.^[1]^ The band gap of Heusler family compounds can be readily tuned from 0 to 4 eV by carefully selecting the chemical composition.[^1^, ^2^, ^3^] These result in distinctly different electrical transport behaviors from highly conductive metals to insulators. More interestingly, one can even predict and then design the magnetic states of Heusler compounds as their magnetic ordering is strongly dependent on the arrangement of the atoms.^[4]^ Taking half‐metallic Heusler alloys as an example, the spin channel filling is related to the number of valence electrons. Thereby, the spin magnetic moment can be obtained according to the Slater‐Pauling rule in which the number of valence electrons is solely involved.^[5]^

For heterogeneous catalytic reactions such as electrochemical water splitting, high‐efficient and economic catalysts based on non‐noble metals are required for the scalable hydrogen production. This is particularly important for the oxygen evolution reaction (OER) at the anode, which is thermodynamically sluggish and limiting the overall efficiency of water electrolysis.^[6]^ Design of OER electrocatalysts with low overpotential has been an interesting topic to both fundamental research and industrial applications under the framework of hydrogen economy.^[7]^ For OER catalysis, the adsorption of various reaction intermediates on the surface of catalysts is a key step. The adsorption behaviors are governed by the electronic structures of the investigated catalyst.^[8]^ The Shao‐Horn group reported the correlation between intrinsic activity and *e*_g_ orbital filling of transition metal cations in a series of perovskite catalysts.^[9]^ A universal principle was thus put forward using *e*_g_ orbital filling as an activity descriptor, namely the Shao‐Horn (SH) principle. It was proposed that *e*_g_ orbital filling influences the binding energy of oxygen intermediates to the catalyst surface, and as a result alters the OER activity. Accordingly, SH principle points out a rational way to optimize OER catalysts, which is based on tuning the *e*_g_ orbital filling of metal sites.^[10]^ So far, most reported works adopted a rather similar strategy of introducing vacancies through heteroatom doping.[^8^, ^11^] Although it has been demonstrated to be effective to regulate *e*_g_ electron configuration of various transition metal compounds, a new class of materials, like Heusler compounds, with precisely controlled *e*_g_ orbital filling is a stimulating platform for further development of OER catalysts.

Intermetallic compounds with transition metals as the constituents have attracted increasing attention for the discovery of new catalysts and the investigation of catalytic mechanisms. In this regard, Heusler compounds could be a potential new platform which however was rarely noticed in the catalysis community. Until recently, Kojima et al. reported selective hydrogenation of alkynes over Co_2_MnGe and Co_2_FeGe, demonstrating the potential of Heusler compounds as new catalysts.^[12]^ Interestingly, the metallic conductivity and multi‐metal constituent of Heusler compounds resemble high entropy alloys that were discovered as promising OER catalysts several years ago.[^13^, ^14^] Therefore, Heusler compounds with designed tunability of *e*_g_ electron configuration, metallic conductivity, and multi‐metal constituent, show great potential as a new class of catalyst for OER.

In this work, to the best of our knowledge, we report the first study of Heusler compounds as novel electrocatalysts towards the OER. Heusler compounds (Co_2_Mn*Z*) with high crystallinity and homogeneous element distribution were prepared by the arc‐melting method. The *e*_g_ electron configuration of reactive Co sites was precisely regulated by varying unreactive metal sites (*Z*=Ti, Al, Ga, V). A solid correlation between the *e*_g_ orbital filling of reactive sites and OER activity was found on Co_2_Mn*Z* compounds whereby higher catalytic current was achieved on the catalyst when *e*_g_ orbital filling approaches to unity. Furthermore, another set of Heusler compounds (Co_2_V*Z*) were prepared and their OER activity demonstrates the volcano‐shaped dependence on *e*_g_ orbital filling of reactive Co sites.

Co_2_ *YZ* Heusler compounds are crystallized in the cubic structure *Fm* $\bar{3}$ *m* (space group no. 225) with Cu_2_MnAl (L2_1_) as a prototype. As exhibited in Figure 1 a, Co, *Y*, and *Z* atoms occupy the Wyckoff position 8c (1/4 , 1/4 , 1/4 ), 4b (1/2 , 1/2 , 1/2 ), and 4a (0, 0, 0), respectively. The electrical and magnetic properties of Heusler compounds can be simply connected to their valance electron number *N*_v_. Generally, Heusler compounds with *N*_v_ of 24 show a semiconducting behavior, where compounds with a larger or smaller *N*_v_ exhibit metallic behavior and magnetism. The magnetic moment *M* of Co_2_ *YZ* Heusler compounds with four atoms per unit cell follows the Slater‐Pauling rule, i.e., *M*=(*N*_v_‐24) *μ*_B_. The molecular orbital diagram for Co_2_ *YZ* Heusler compounds is presented in Figure 1 b by taking Co_2_MnAl (*N*_v_=28) as an example. The atomic *d* orbitals of [Co*Z*] ([CoAl]) substructure and the second Co atom built two sets of *t*_2g_ and *e*_g_ hybrid orbitals. *Y* (Mn), which occupies the octahedral lattice site, adds its *d* states between these hybrid states. For Co_2_MnAl, 24 valence electrons doubly occupy the orbitals, resembling the configuration of semiconducting Fe_2_VAl (*N*_v_=24), the additional 4 valence electrons singly occupy the *e*_g_ orbitals with parallel spin orientation owing to the small energy difference between the orbitals. This results in a half‐metallic state and a *M* of 4 μ_B_ per formula unit. Similarly, Co_2_VGa, Co_2_VSn, Co_2_MnGa, Co_2_MnTi, and Co_2_MnV, which have *N*_v_ of 26, 27, 28, 29, and 30, also show half‐metallic states with *M* of 2, 3, 4, 5, and 6 μ_B_ per formula unit, respectively (Figure 1 c). As the Co sites are in high spin states for all the investigated Heusler compounds, we are able to obtain the values of the local moment and *e*_g_ filling correspondingly (see Table S1 in the Supporting Information). More importantly, both the local magnetic moment and *e*_g_ filling of Co sites are not crystal surface‐dependent (except the very first layer). This suggests a well‐defined *e*_g_ orbital structure of Heusler compounds, making them a good platform for investigating the effect of electronic occupation in *e*_g_ orbitals on their catalytic performances.

**Figure 1:**
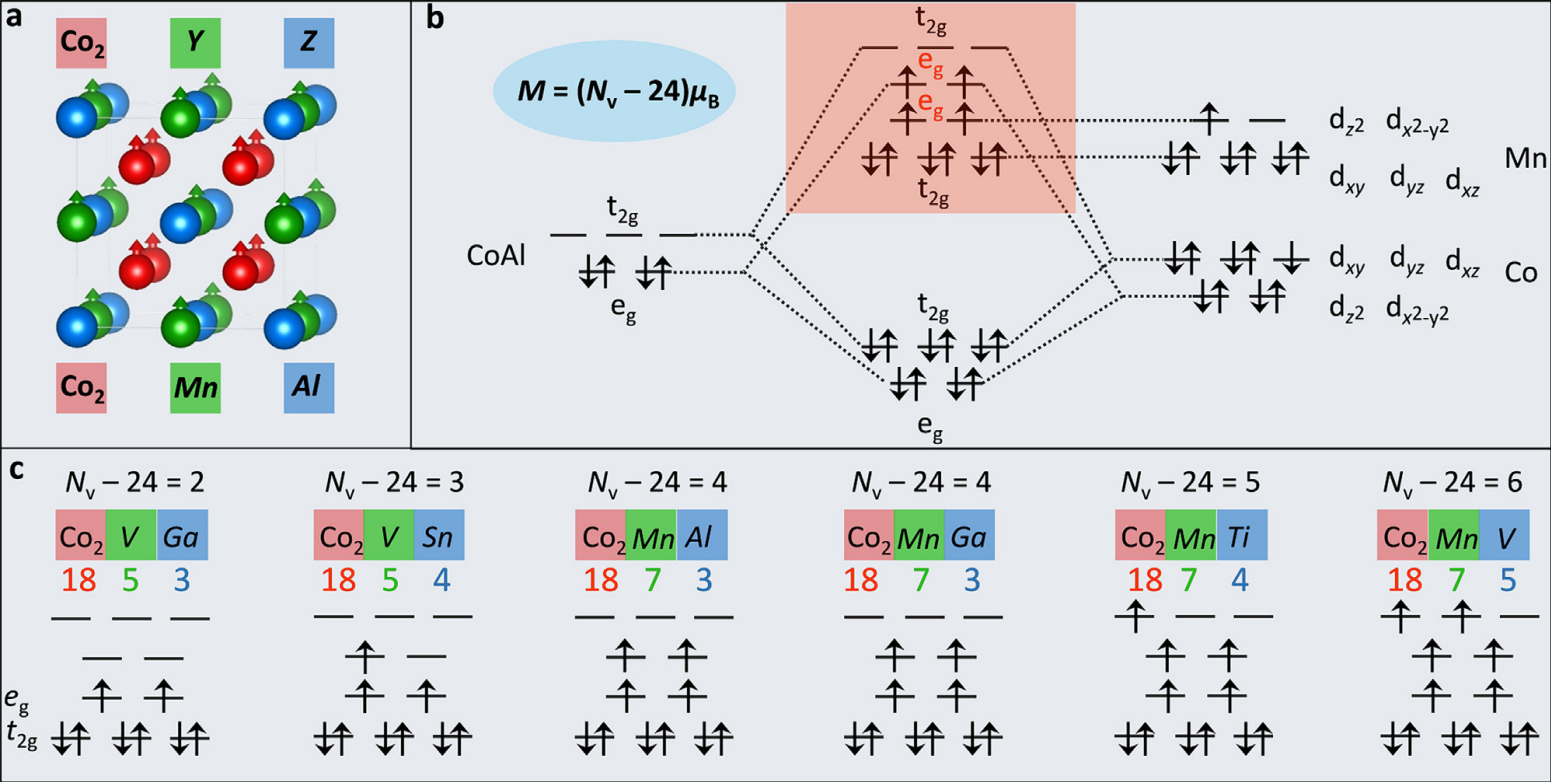
a) Crystal structure of Co_2_ *YZ* Heusler compounds. The magnetic moment *M* of Co_2_ *YZ* can be calculated according to the Slater‐Pauling rule: *M*=(*N*_v_‐24) *μ*_B_. b) Illustration of molecular orbital diagram of Co_2_MnAl. c) The magnetic moment and electron occupation of the six selected Co_2_ *YZ* compounds. For simplicity, only the part (light red region) which presents different electron occupation is shown. The counting of valence electron number *N*_v_ is shown at the top.

Here, we selected Co_2_‐based Heusler compounds (Co_2_Mn*Z*), i.e., Co_2_MnAl, Co_2_MnTi, Co_2_MnGa, and Co_2_MnV, with different *N*_v_ and *e*_g_ filling, for OER study. It is not only due to the fact that Co as a 3d metal possesses varying *e*_g_ electron configuration with introducing different neighboring atoms, but also because of the high OER activity that Co‐based catalysts have shown in the previous study.[^15^, ^16^, ^17^, ^18^] All the compounds were first synthesized by arc‐melting method into bulk crystals (Figure S1), which can be later cut into desired shapes.^[2]^ As seen in Figure 2 a–c, Co_2_MnGa crystal was cut into a cuboid shape with the dimension of 0.5×0.5×8 mm^3^ that shows rough surfaces. Additionally, its mechanic robustness and metallic conductivity provide the great potential of such high‐quality crystals in various applications including in electrocatalysis.

**Figure 2:**
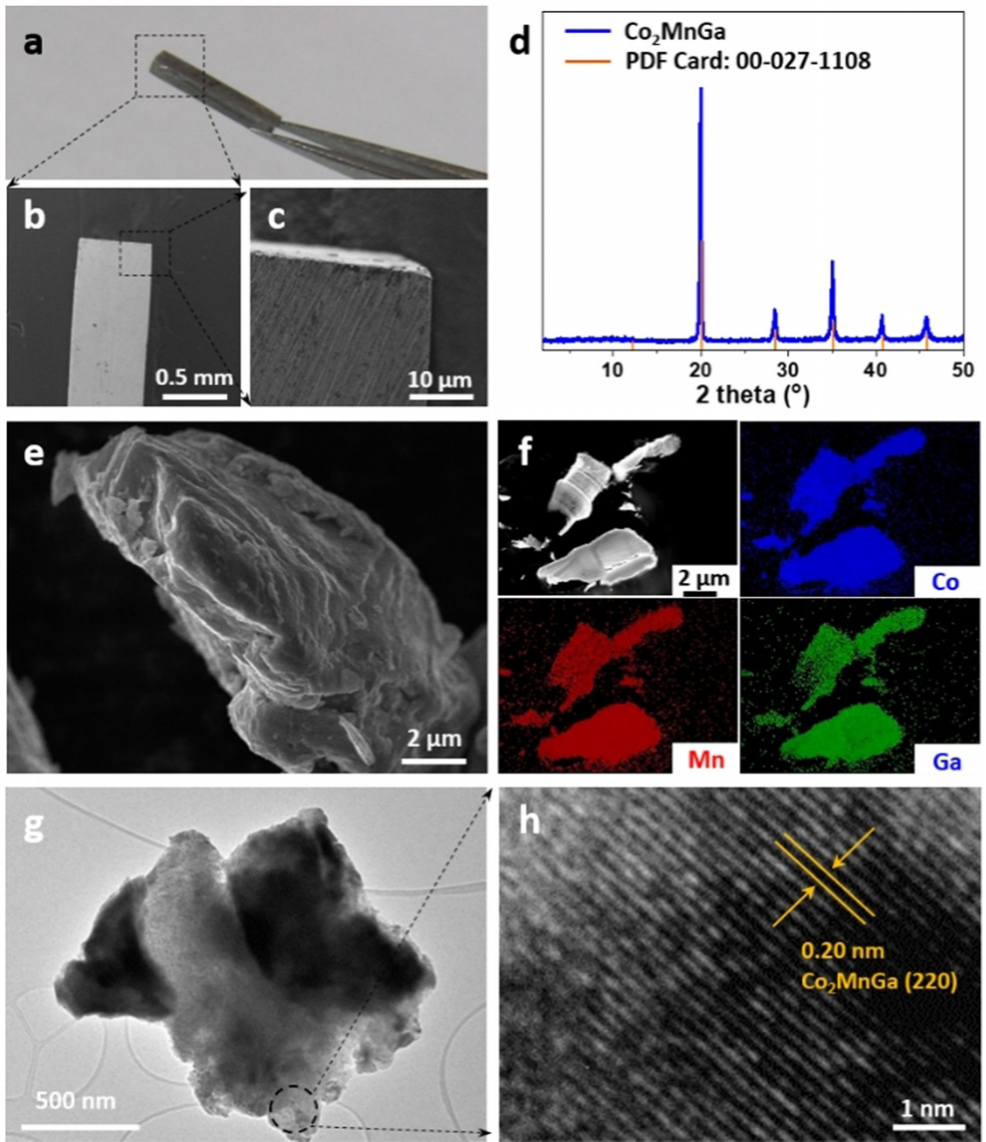
a) Digital image of Co_2_MnGa crystal held by a tweezer. b,c) Low‐resolution SEM images of Co_2_MnGa single crystal. d) XRD pattern, e) SEM, f) elemental mapping, g) TEM, and h) HR‐TEM images of powdered Co_2_MnGa after ball‐milling treatment.

For detailed structural characterizations, the large crystals were treated with mechanical ball milling to form fine powders. X‐ray diffraction (XRD) analysis was first employed to characterize the crystal structure of Heusler compounds. As shown in Figure 2 d, powdered Co_2_MnGa exhibits distinct reflections centered at 20.0°, 28.5°, 35.0°, 40.7°, and 45.8° (2 theta value), corresponding to (220), (400), (422), (440), and (620) facets of Co_2_MnGa cubic structure (PDF card: 27‐1108). The other compounds are also highly crystalline as demonstrated by the XRD patterns (Figure S2). It is worth mentioning that Co_2_VSn and Co_2_MnTi are not phase‐pure, probably due to the impurities introduced by the processes of arc melting and ball‐milling. This can be also seen from the elemental compositions of powdered Heusler compounds, as determined by energy‐dispersive X‐ray (EDX) spectroscopy. As shown in Figure S3, a small amount of impurity elements like Si, Fe, and Cr (≈2 at. %) were found on each sample, as typically introduced by the synthesis procedures. Nevertheless, the major compositions are Co, Mn and *Z* (Ti, Al, V, Sn), and the atomic ratio of Co: Mn: *Z* is in good agreement with the stoichiometry in Co_2_Mn*Z* compounds, as seen in Table S2.

Electron microscopy was then conducted to visualize the surface morphology of powdered Heusler compounds. As shown in the scanning electron microscopy (SEM) images (Figure 2 e and Figure S4), large crystals up to size of centimeters were milled into aggregates in the scale of micrometer. Elemental mapping images revealed the uniform distribution of Co, Mn, and *Z* (Ti, Al, V, and Ga), suggesting the high quality of Heusler crystals (Figure 2 f and Figure S5–S7). A careful cutting on Co_2_MnGa crystal allowed for direct imaging on the internal structure. As shown in Figure S8, single crystals were not grown into impermeable solid, instead, porosity was formed inside the crystals, in agreement with the observed rough surface in Figure 2 c. Furthermore, transmission electron microscopy (TEM) images were taken for a closer observation of the crystal, as seen in Figure 2 g and 2 h. An overview TEM image shows that the average size of crystal domains is below 2 μm (Figure S9). A representative crystal domain in Figure 2 g is an aggregate of smaller crystalline particles. The observed lattice fringes in the high‐resolution TEM image (Figure 2 h) show a spacing of 0.20 nm, corresponding to the (220) planes of Co_2_MnGa crystal. Moreover, spot EDX spectrum was collected to determine the local elemental ratio (Figure S10). The atomic ratio of Co : Mn : Ga is measured as 2 : 1 : 1, perfectly matching with the stoichiometry in the Co_2_MnGa compound.

To investigate the electrocatalytic activity of Heusler compounds, Co_2_Mn*Z* powder samples were deposited on glassy carbon (GC) electrode, and their OER activities were measured following the protocol proposed by the Jaramillo group.^[6]^ Linear sweep voltammetry (LSV) was conducted to collect the polarization curves of Co_2_Mn*Z* compounds. As shown in Figure 3 a, the catalytic activity of Co_2_Mn*Z* compounds has an obvious dependence on the *Z* element. Among them, Co_2_MnTi delivered the highest current density. Similar activity was obtained with Co_2_MnAl and Co_2_MnGa, while the lowest current was achieved on the compound when *Z* is vanadium. The same trend could be observed on the corresponding Tafel slopes. In Figure 3 b, Co_2_MnTi as the most active sample showed the lowest value of Tafel slope (63 mV dec^−1^), suggesting a more favorable catalytic kinetics. Almost the same Tafel slopes were obtained on Co_2_MnAl (68 mV dec^−1^) and Co_2_MnGa (67 mV dec^−1^) compounds. A typical value of Tafel slope of ≈60 mV dec^−1^ is generally reported for Co based catalysts where oxidized Co species determine the OER kinetics.[^19^, ^20^, ^21^] Similar Tafel slopes are observed on these Heusler compounds (63–68 mV dec^−1^) indicates that Co sites catalyze the OER process.

**Figure 3:**
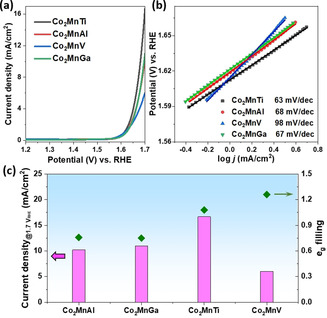
a) The LSV curves of Heusler compounds (Co_2_Mn*Z*, *Z*=Ti, Al, V, and Ga). The current density was determined by the geometry surface area of the GC electrode (0.196 cm^2^). b) Tafel slopes derived from the LSV curves in (a) correspondingly. c) Comparison of current density at 1.7 V vs. RHE and *e*_g_ electron filling for Co_2_MnZ compounds. The current density was determined by the geometric surface area of the GC electrode (0.196 cm^2^).

In addition, long‐term stability of the Heusler compound was evaluated by applying a voltage of 1.63 V vs. RHE continuously for 12 h. As shown in Figure S11, Co_2_MnGa immobilized on carbon fiber paper as anode delivered a current density at around 2.5 mA cm^−2^ with a continues increasement over 12 h. The electrode was further checked by SEM before and after stability test. As shown in Figure S12 and S13, Co_2_MnGa aggregates were robustly immobilized on electrode with the use of Nafion as a binder. A significant amount of Ga was leached out during measurement while Co and Mn were more resistant to the KOH electrolyte solution. In order to study alteration of the surface structure, we conducted a X‐ray photoelectron spectroscopy (XPS) study on Co_2_MnGa before and after electrochemical test. In Figure S14, the survey of the XPS spectra indicated a similar chemical composition except that Ga was not detected on the surface after the electrochemical test. This is further supported by the disappeared peak in Ga 2p region (Figure S15c), which demonstrated surface leaching of Ga under applied voltage in alkaline electrolyte. On the other hand, Co and Mn were more resistant to electrolysis conditions. Nevertheless, significant oxidation was observed along with the formation of oxyhydroxide species on the surface during OER (Figure S15, a more detailed discussion is provided in supporting information). Therefore, the increasement of current density could be attributed to surface oxidation/amorphization in alkaline electrolyte.[^13^, ^14^] Furthermore, we are able to fabricate an electrode using a cuboid Co_2_MnGa crystal directly. As seen in Figure S16, a single crystal of Co_2_MnGa is connected with inactive Ti wire by silver paste. For a small electrode (0.5×0.5×8 mm^3^), a substantial current (>15 mA) was achieved at 1.7 V vs. RHE, demonstrating its potential as a practical electrode for efficient water electrolysis.

As mentioned, *Z* element influences the *e*_g_ electron configuration of Co through the covalent bonding, leading to precise regulation of *e*_g_ orbital filling. Considering the fact that both Mn and *Z* elements are not preferred active sites, Co sites are more efficient OER catalyst and in this work are key contributors to OER activity. Thus, we could correlate the OER activity of Co_2_Mn*Z* compounds with the *e*_g_ orbital filling of reactive Co. As shown in Figure 3 c, the catalytic performance of Co_2_MnAl and Co_2_MnGa can be associated with the values of the *e*_g_ filling, which are 0.76 and 0.75, respectively. When the *e*_g_ filling approached to unity (1.08), superior OER activity is achieved with Co_2_MnTi. Further increasement of the *e*_g_ filling is not beneficial for OER catalysis, as illustrated by the lowest catalytic current delivered by Co_2_MnV catalyst (*e*_g_ filling: 1.26). Obviously, the OER activity of Heusler compounds shows a strong dependence on the *e*_g_ filling of reactive metal species.

To verify this dependence, another set of Heusler compounds (Co_2_V*Z*, *Z*=Sn and Ga) were synthesized following the same method. Detailed characterization confirms the good quality of Co_2_V*Z* crystals and their similar physical properties including surface morphology and domain size, as shown in Figure S17–S20. Employed as OER catalysts, Co_2_VSn is slightly more active with reaching a current density of 16.6 mA cm^−2^ at 1.7 V vs. RHE, in comparison to Co_2_VGa (15 mA cm^−2^, Figure S21a). A reasonable Tafel slope (65 mV dec^−1^) is shown on both Co_2_VGa and Co_2_VSn compounds (Figure S21b). Figure 4 summarizes the OER activity of Co_2_Mn*Z* and Co_2_V*Z* compounds, with plotting current density against the *e*_g_ orbital filling of reactive Co. A volcano‐shaped curve was obtained, further supporting the dependence of OER activity on the *e*_g_ filling of reactive metal species in Heusler compounds. Moreover, we normalized the catalytic activity by the electrochemical surface area (ECSA) to compare the intrinsic activity of these Heusler compounds. As shown in Figure S22–23, a small value of double‐layer capacitance (C_dl_; 2.4–3.2 μF) was obtained on all the Heusler compounds. Correspondingly, all the electrodes have a small ECSA (0.06–0.08 cm^2^) due to the large particle size of crystals, in good agreement with the observation from electron microscopy. Upon normalization to ECSA, a volcano‐shaped relation was also obtained between intrinsic OER activity and the *e*_g_ orbital filling of reactive Co (Figure S24), which reveals the regulation role of *e*_g_ orbital filling on Heusler catalysts.

**Figure 4:**
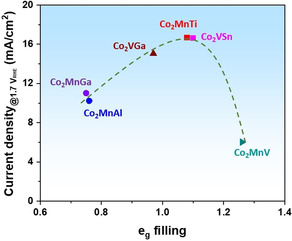
The volcano‐shape plot of the OER catalytic activity, defined by the current density at 1.7 V vs. RHE, against the occupancy of the *e*_g_ electron of Co in Heusler compounds (Co_2_Mn*Z* and Co_2_V*Z*).

It is widely accepted that the intrinsic activity of an OER catalyst is determined by the binding energy between surface metal sites and oxygen intermediates (e.g. *OH, *O, *OOH, and *O_2_).[^22^, ^23^, ^24^] Regulation on the *e*_g_ filling of surface metal ions could contribute to a more balanced binding energy and thus enhance OER activity, as proposed in SH principle and demonstrated by both experimental results and theoretical calculation in recent studies.[^9^, ^11^, ^25^, ^26^] As demonstrated in this study, precise regulation of the *e*_g_ filling of Co sites can be achieved through tuning the elemental compositions of Co_2_ *YZ* Heusler compounds. A balanced adsorption/desorption bonding of oxygen intermediates is expected on optimized Heusler compounds with the *e*_g_ filling of Co sites approaching to unity. Resultantly, Co_2_MnTi, Co_2_VSn, and Co_2_VGa with *e*_g_ filling close to 1 are more efficient OER catalysts.

Furthermore, we studied the adsorption behavior of oxygen intermediates at the surface of Co_2_VSn that exhibited the best OER activity among all the investigated Heusler compounds. Figure S25a depicts a general mechanism of OER on transition metal‐based catalysts, in which the formation of *OOH on metal sites is a crucial step.^[24]^ Our theoretical calculation result suggests an unstable *OOH intermediate on the surface Co sites (Figure S25b). Instead of coupling with hydroxyl anion to generate oxygen, *OOH intermediate is immediately decomposed into *OH and *O. This indicates that a preferable OER pathway on Heusler compounds is via a direct combination of two M‐O species with forming oxygen product and meanwhile releasing active sites. Thus, M−O bonding strength plays an important role on governing the overall OER kinetics on Heusler catalysts. For transition metals in the octahedral crystal field, the splitting of *d*‐orbitals results in the formation of three *t*_2g_ and two *e*_g_ orbitals. Among which, only the *e*_g_ orbitals have a density of states that is out of the plane (Figure S25c), which enables an overlap with the O *2p* orbitals of oxygen intermediates (Figure S25d). As a result, tuning the *e*_g_ orbitals of Co sites could effectively modulate adsorption energy as well as electron transfer between surface cations and adsorbates towards a more efficient OER catalyst.[^9^, ^27^, ^28^]

In conclusion, high‐quality crystals of Heusler compounds (Co_2_Mn*Z*, *Z*=Ti, Al, V, and Ga) were prepared by arc‐melting method and were used as a new platform for electrochemical oxygen evolution reaction. The comparison on the OER activity illustrates a strong correlation with the *e*_g_ orbital filling of reactive Co sites and the optimal catalytic current was achieved when *e*_g_ orbital filling approached to unity. This was further supported by another set of Heusler compounds (Co_2_V*Z*, *Z*=Sn and Ga). Overall, the OER activity of Heusler compounds demonstrates a volcano‐shaped dependence on *e*_g_ orbital filling of reactive transition metal cations. The theoretical calculation suggested a preferable OER pathway on Heusler compounds via a direct combination of two M‐O species. Thus, tuning *e*_g_ orbital filling is an effective strategy to modulate the M−O bonding strength towards more active OER catalysts. This study not only explores the potential of Heusler compounds as novel OER electrocatalysts but also illustrates the optimization of catalysts based on precise regulation of electron configuration.

## Conflict of interest

The authors declare no conflict of interest.

## Supporting information

As a service to our authors and readers, this journal provides supporting information supplied by the authors. Such materials are peer reviewed and may be re‐organized for online delivery, but are not copy‐edited or typeset. Technical support issues arising from supporting information (other than missing files) should be addressed to the authors.

Supplementary
