# Supplementary material for: Tunable e g Orbital Occupancy in Heusler Compounds for Oxygen Evolution Reaction
Source: Angew Chem Int Ed Engl. 2021 Feb 3;60(11):5800–5. doi: 10.1002/anie.202013610 (PMC7986729; doi:10.1002/anie.202013610)
Supplement: Supplementary file 1 — Supplementary [file ANIE-60-5800-s001.pdf]

Angewandte Chemie
Eine Zeitschrift der Gesellschaft Deutscher Chemiker

Supporting Information

Tunable e_g Orbital Occupancy in Heusler Compounds for Oxygen Evolution Reaction**

Mingquan Yu[+], Guowei Li[+], Chenguang Fu[+], Enke Liu, Kaustuv Manna, Eko Budiyanto, Qun Yang, Claudia Felser, and Harun Tüysüz**

anie_202013610_sm_miscellaneous_information.pdf

Experimental Section

Synthesis of Heusler compounds.

Polycrystalline ingots of Heusler alloys studied in this work were synthesized by arc melting stoichiometric amounts of the constituent high-purity elements (99.999 %) in an arc furnace with a water-cooled Cu hearth under an Ar atmosphere. Deailed synthesis procedure can be found elsewhere.[1] To increase the volume homogeneity, all the ingot were remelted more than two times. The final weight loss of the samples was less than 0.5% of the initial weight.

For the synthesis of bulk single crystals, the induction-melted samples were crushed into fine powders and then packed in a custom-designed sharp-edged alumina tube that was sealed in a tantalum tube. Take the growth of Co_2MnGa crystal as an example, the tube was heated to 1523 K and soaked for 10 h to ensure homogeneity of the melt and then slowly cooled to 1023 K.

Irregularly shaped crystals of Heusler compounds for electrochemical studies (Figure S1) were crushed into fine powders through a ball milling process. Each sample was processed in a planetary ball mill with keeping a rotating speed at 500 rpm for 10 minutes.

Electrochemical Measurements.

Electrochemical measurements were performed in a three-electrode configuration using a rotating disc electrode (Model: AFMSRCE, PINE Research Instrumentation). A hydrogen reference electrode (HydroFlex, Gaskatel) and Pt wire were used as reference electrode and counter electrode, respectively. All measurements were carried out in 1 M KOH electrolyte in a Teflon cell. The temperature of the cell was controlled at 25 ^oC using a water circulation system. Prior to the electrochemical measurement, argon was purged through the cell for 30 min to remove oxygen from the electrolyte. During all measurements, argon was continuously purged to remove generated oxygen. Working electrodes were fabricated by depositing target materials on glass carbon (GC) electrodes (PINE, 5 mm diameter, 0.196 cm^2 area). Before use, a thorough cleaning was done on the surface of GC electrodes by polishing with Al_2O_3 suspension (5 and 0.25 μm, Allied High Tech Products, Inc.). Working electrodes were fabricated by drop-casting catalyst ink on GC electrodes. In detail, 4.8 mg of powder sample was first dispersed in a mixed solution containing 0.75 mL of H_2O, 0.25 mL of 2-propanol (Aldrich, 99.5 %) and 50 μL of Nafion (5% in a mixture of water and alcohol). Afterward, the mixture solution was immersed in a sonication bath for 30 min to form a homogeneous ink. Finally, 5.25 μL of catalyst ink was dropped onto the GC electrode and dried under argon atmosphere for 30 min. The catalyst loading on GC electrodes was calculated to be around 0.12 mg/cm^2 following this procedure. For the stability test, an

electrode was fabricated by dropping 110 μL on a carbon fiber paper (1 cm × 1 cm), with a catalyst loading of around 0.5 mg/cm^2.

After dipping GC electrodes into KOH electrolyte, the linear sweep voltammetry (LSV) curves were collected by sweeping the potential from 0.7 V to 1.7 V_{RHE} with a scan rate of 10 mV/s. To minimize the effect of the generated oxygen bubble, a rotating disc electrode configuration was kept a rotation speed of 2000 rpm. The IR drop was compensated at 85 % automatically *via* the potentiostat software (EC-Lab V11.01).

The value of ECSA was determined by measuring the non-Faradaic capacitance current from the scan-rate dependence of CVs. CV scans with increasing scan rates, from 20 to 180 mV/s, were collected in a non-Faradaic region (0.9 - 1 V_{RHE}). By plotting the capacitive current (j_{anode} - $j_{cathode}$) against the scan rate and fitting with a linear fit, the value of C_{dl} can be estimated as half of the slope. The ECSA of each sample was calculated according to this equation: ECSA = C_{dl}/C_s, where C_s is the specific capacitance. In this work, 0.04 mF/cm^2 was chosen as the reference value for the measurements in 1M KOH solution.[2]

Characterization. Powder X-ray diffraction (XRD) patterns of Co_2MnX compounds were collected on a Stoe STADI P transmission diffractometer equipped with a primary Ge (111) monochromator (Mo$K\alpha_1$) and a position-sensitive detector. For Co_2VX compounds, their XRD patterns were collected on a STOE theta/theta diffractometer in Bragg-Brentano geometry (Cu $K\alpha_{1/2}$ radiation) with a secondary monochromator. Low-resolution scanning electron microscopy (SEM) images of cuboid crystal were recorded with a Hitachi TM3030. Transmission electron microscopy (TEM) images of powder samples were measured at 100 kV by an H-7100 electron microscope from Hitachi. High-resolution TEM (HR-TEM) and scanning electron microscopy (SEM) images were taken on HF-2000 and Hitachi S-5500 microscopes, respectively. To slice Co_2MnGa crystal, Co_2MnGa podwer was first embedded in Spurr resin and then trimmed using an EM TRIM milling system (Leica). Thin slices were cut from the resin blocks by microtome with a 35° diamond knife (Reichert Ultra-Cut), dispersed in Milli-Q water, transferred from the water surface on lacy carbon film-coated Cu grids and observed on Hitachi S-5500 (Hitachi) microscope.

X-ray photoelectron spectroscopy (XPS) measurements were conducted on Co_2MnGa/carbon fiber paper before and after electrochemical test *via* a SPECS GmbH spectrometer with a hemispherical analyzer (PHOIBOS 150 1D-DLD). A monochromatized Al Kα X-ray source (E = 1486.6 eV) was employed and operated at 100 W. The base pressure in the analysis chamber was kept at 5 x 10^{-10} mbar during the experiment. The binding energy scale was corrected for surface charging by use of the C 1s peak of contaminant carbon as reference at 284.5 eV.

Theoretical calculation. Our spin-polarized calculations were performed by using the VASP with PAW potentials.[3-4] PBE-GGA was applied to deal with the electron exchange-correlation interaction,[5-7] and DFT-D3 extension of Grimme was adopted to describe the long-range Van der Waals (VdW) interactions between the adsorbate and the substrate. Herein, the (110) plane of

Co_2VSn with the co-expose of Co and V atom was constructed for the adsorption of the adsorbate. A 8×6×1 k-mesh in the BZ was used for the geometry optimization and self-consistent calculations, and the kinetic energy cutoff was set to 400 eV.[7]

Table S1. The Currie temperature (T_c), magnetization (M), and e_g filling of Co-based Heusler alloys (based on theoretical values and our unpublished works).[8-10]

**Compounds**	**T_c (K)**	**M (μ_B)**	**e_g filling (Co moment)**
Co_2MnGa	694	4.05	0.75
Co_2MnAl	685	4.04	0.76
Co_2VGa	357	2.01	0.97
Co_2MnTi	915	4.94	1.08
Co_2VSn	374	3.03	1.1
Co_2MnV	625	5.76	1.26

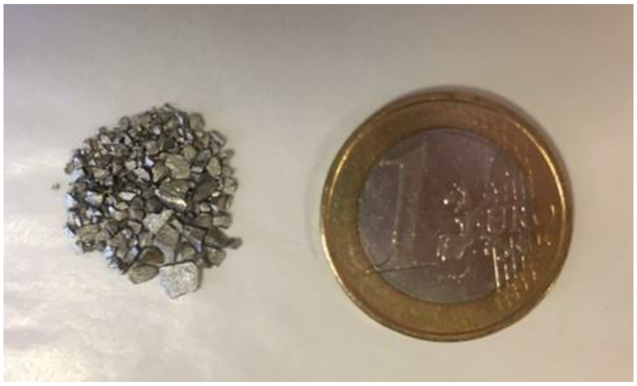

Figure S1. Digital picture of Heusler crystals with a one euro coin as comparison.

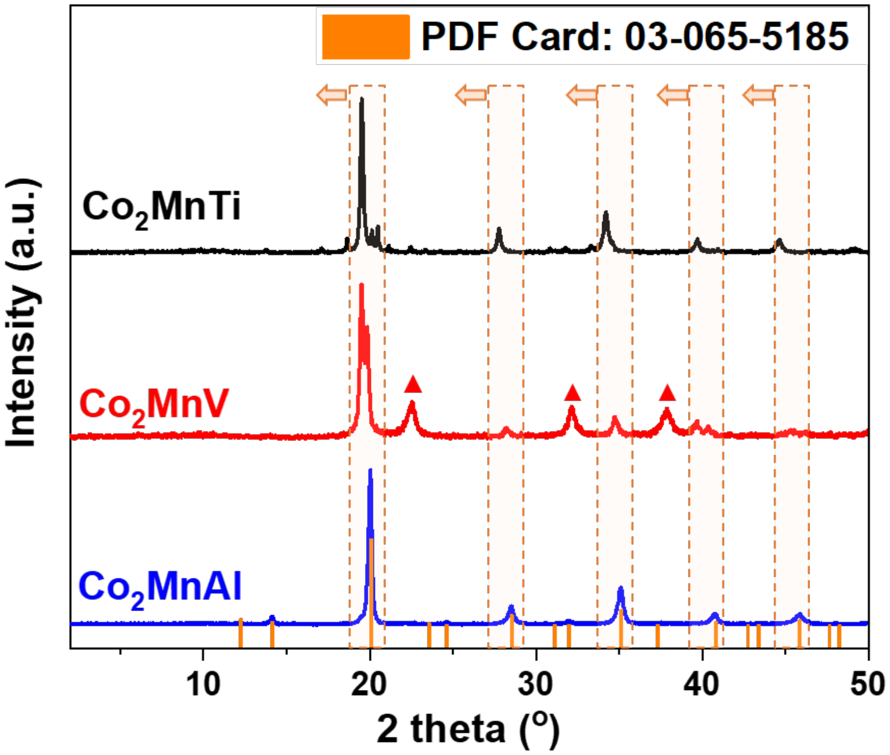

Figure S2. XRD patterns of Co_2MnTi, Co_2MnV, and Co_2MnAl. Orange columns represent the reflection peaks of the cubic Co_2MnAl structure in PDF card: 03-065-5185. Note: a negative shift was shown on the reflections of Co_2MnTi and Co_2MnV, suggesting a lattice expansion in comparison to that of Co_2MnAl. This can be due to the replacement of Al (125 pm) by V (135 pm) and Ti (140 pm) with a larger radius in the cubic structure. In the XRD pattern of Co_2MnV, additional reflections at $2\theta = 22.5^o$, 32.1^o, and 37.8^o, marked by red triangle, match well with the (200), (220), and (311) facets of the Co_3Ti cubic structure (PDF Card: 01-077-7583). EDX result in Figure S3 shows the existence of V instead of Ti. By taking into account that V and Ti have a similar element radius, it is reasonable to postulate that a separate Co_3V phase was formed on Co_2MnV compounds during arc-melting process.

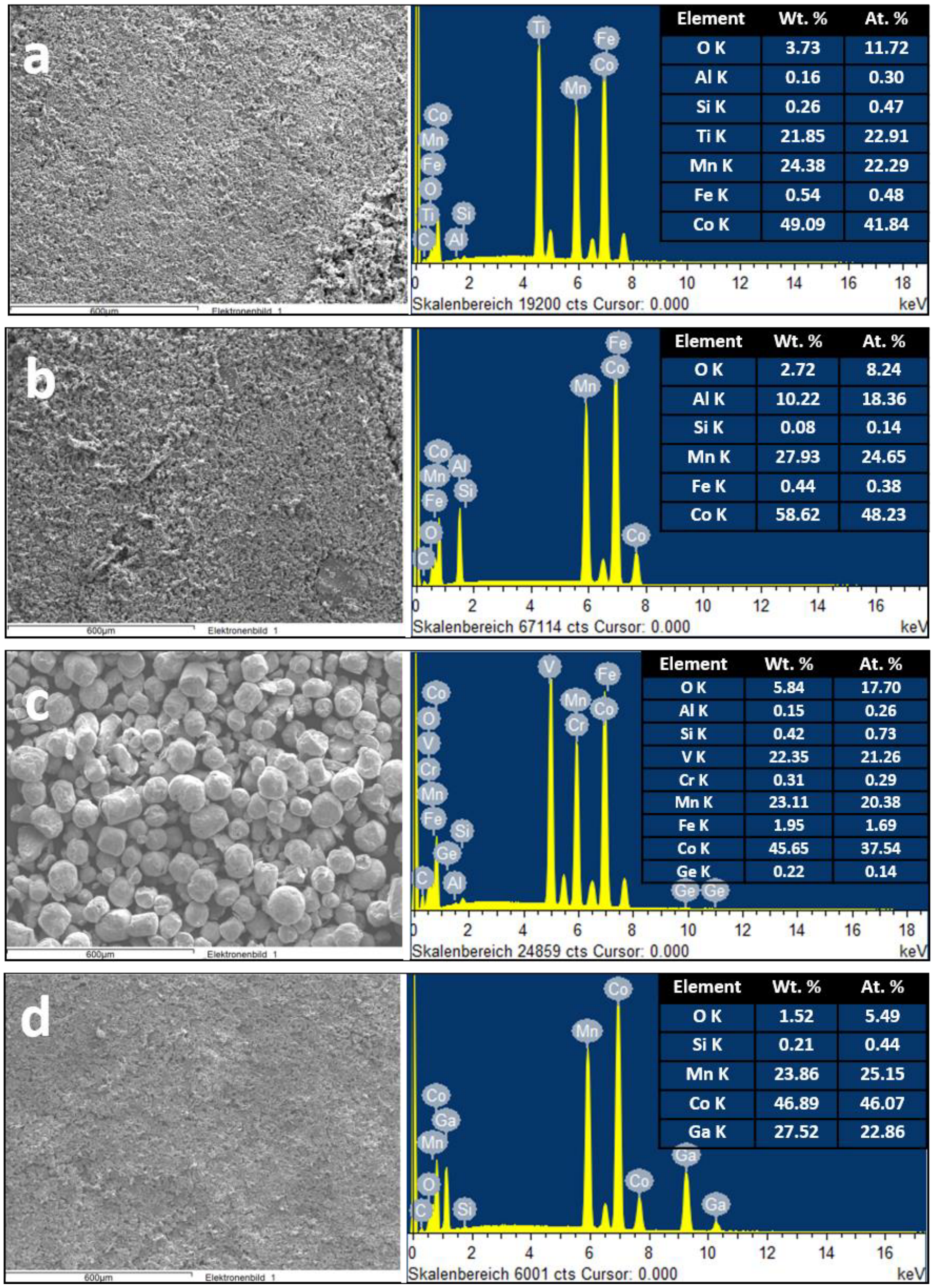

Figure S3. SEM images with corresponding EDX analysis of Heusler compounds: (a) Co_2MnTi, (b) Co_2MnAl, (c) Co_2MnV, and (d) Co_2MnGa.

Table S2. The Co : Mn : X ratio of Co2MnX compounds, derived from EDX results in Figure S3.

	Co_2MnTi	Co_2MnV	Co_2MnAl	Co_2MnGa
Actual ratio of Co : Mn : X	1.8 : 1 : 1	1.8 : 1 : 1	2 : 1 : 0.7	1.8 : 1 : 0.9

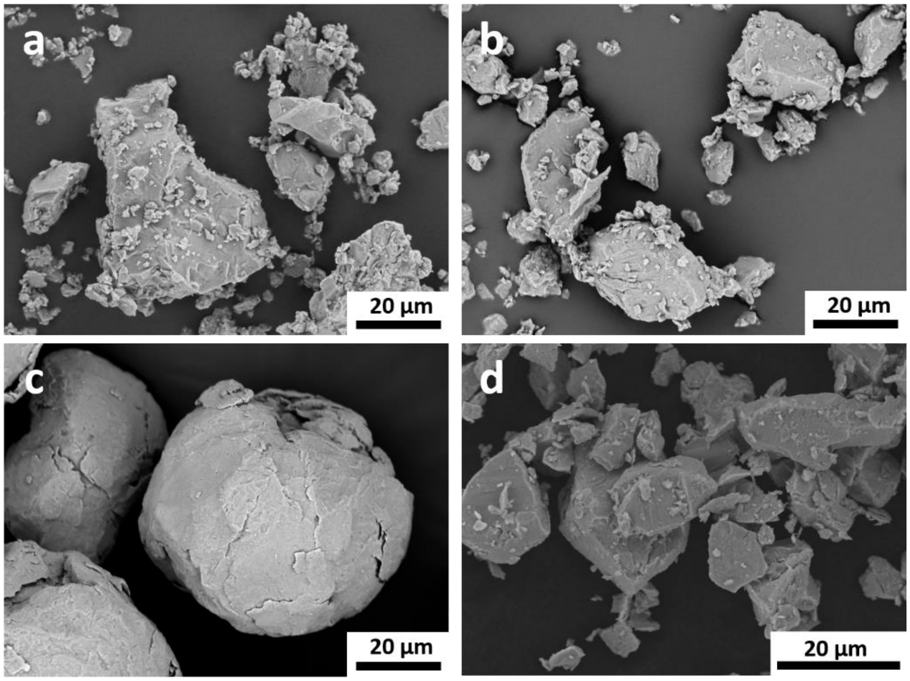

Figure S4. Low resolution SEM images of (a) Co_2MnTi, (b) Co_2MnAl, (c) Co_2MnV, and (d) Co_2MnGa.

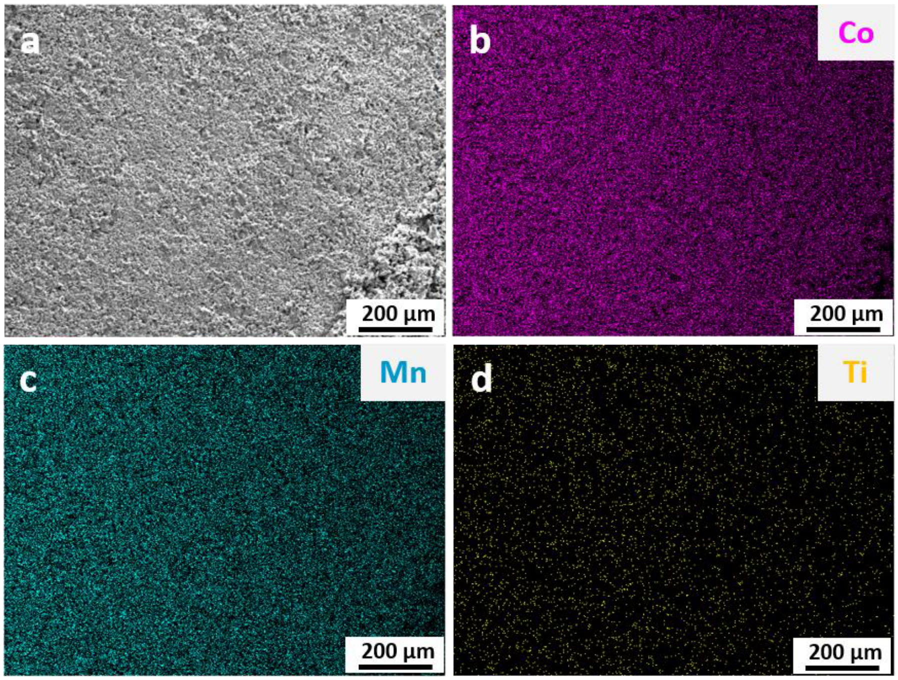

Figure S5. SEM image (a) of Heusler compound Co_2MnTi, and corresponding elemental mapping images of (b) Co, (c) Mn, and (d) Ti.

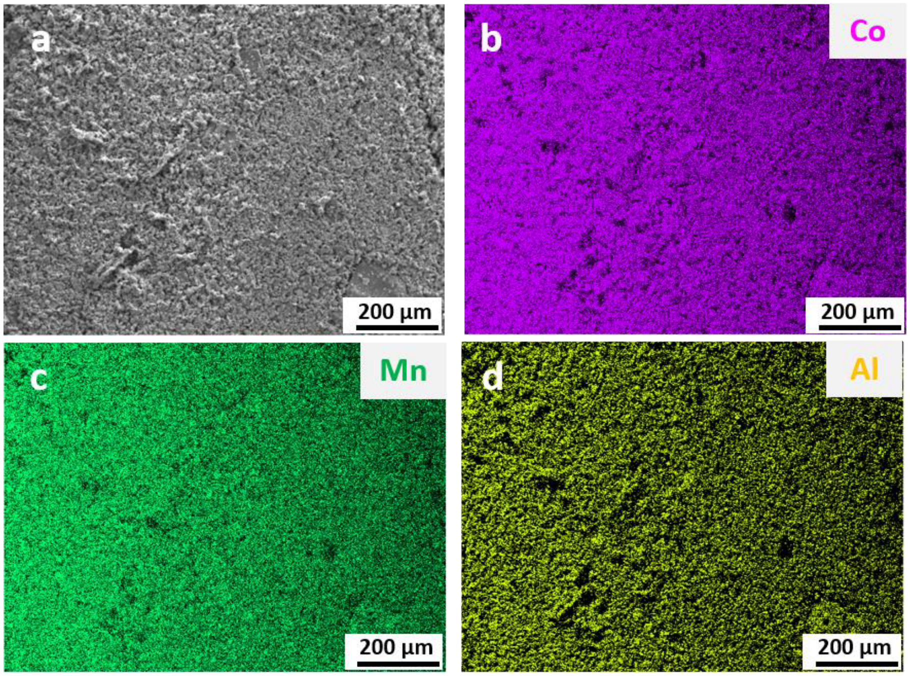

Figure S6. SEM image (a) of Heusler compound Co_2MnAl, and corresponding elemental mapping images of (b) Co, (c) Mn, and (d) Al.

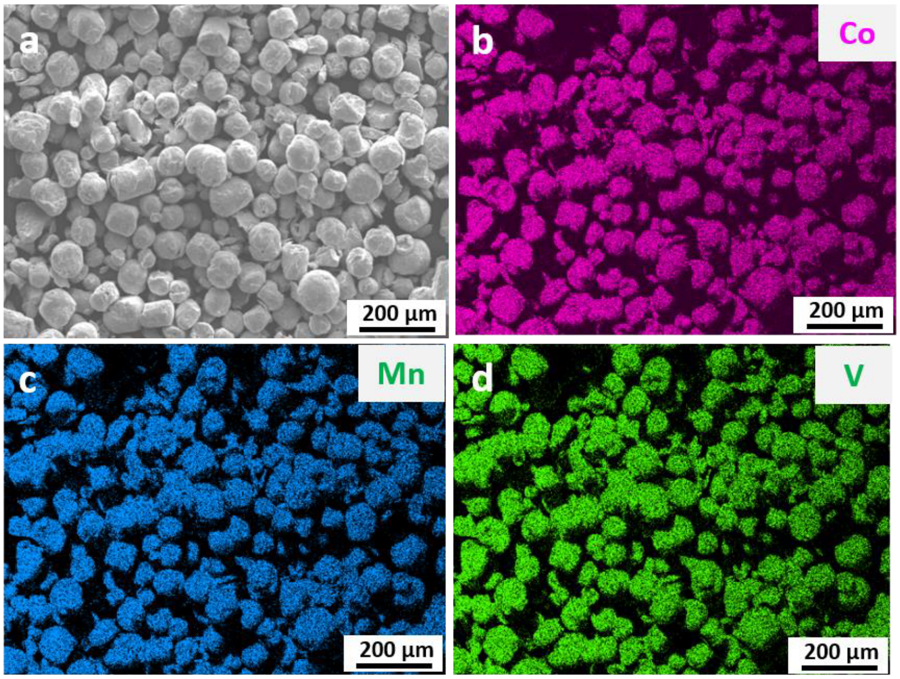

Figure S7. SEM image (a) of Heusler compound Co_2MnV, and corresponding elemental mapping images of (b) Co, (c) Mn, and (d) V.

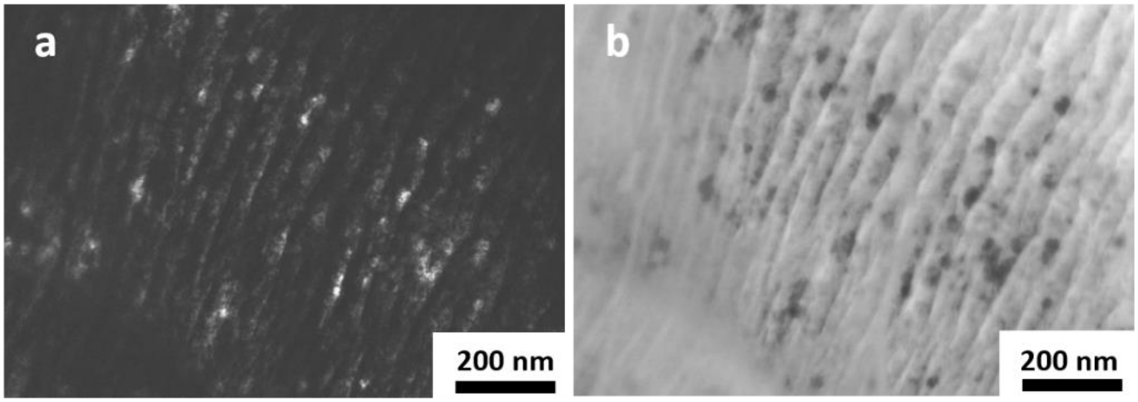

Figure S8. Bright field (a) and dark field (b) STEM images of Co_2MnGa slice. Co_2MnGa crystals were cut into thin sections by a diamond knife (see detailed procedure in experimental section).

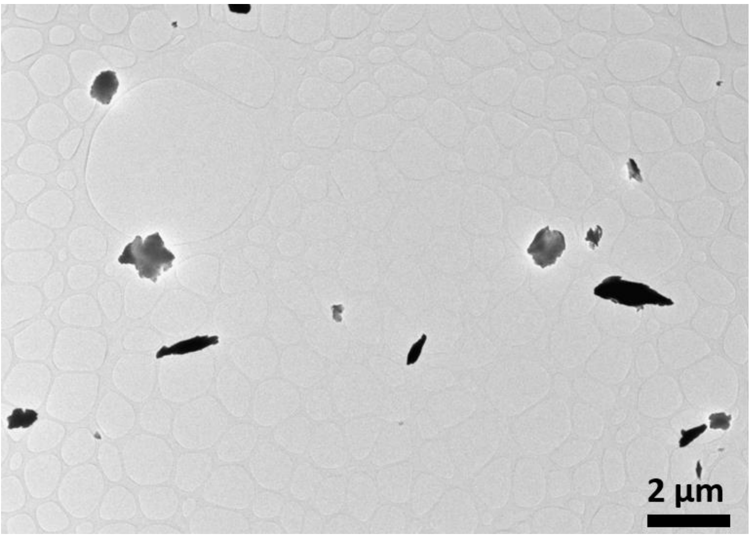

Figure S9. An overview TEM image of Co_2MnGa.

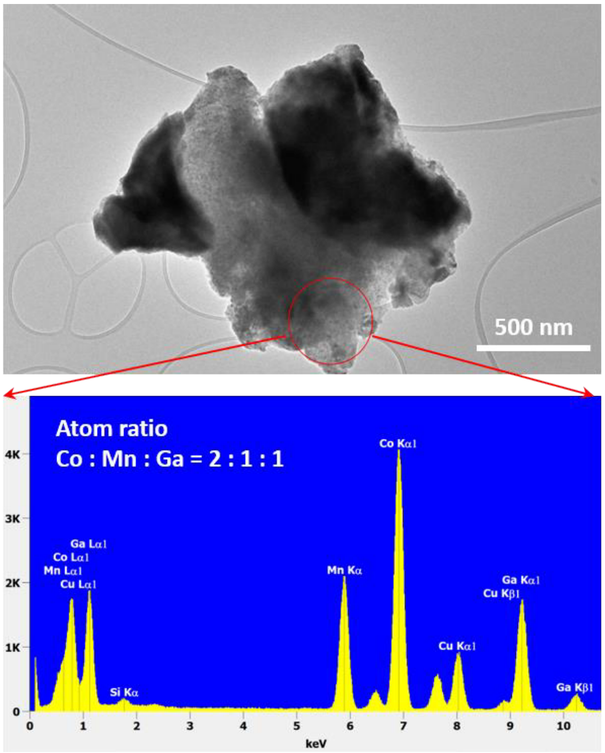

Figure S10. Spot EDX result of Co_2MnGa. The atomic ratio of Co : Mn : Ga was calculated to be 2 : 1 : 1, in perfect agreement with the stoichiometry in Co_2MnGa compound.

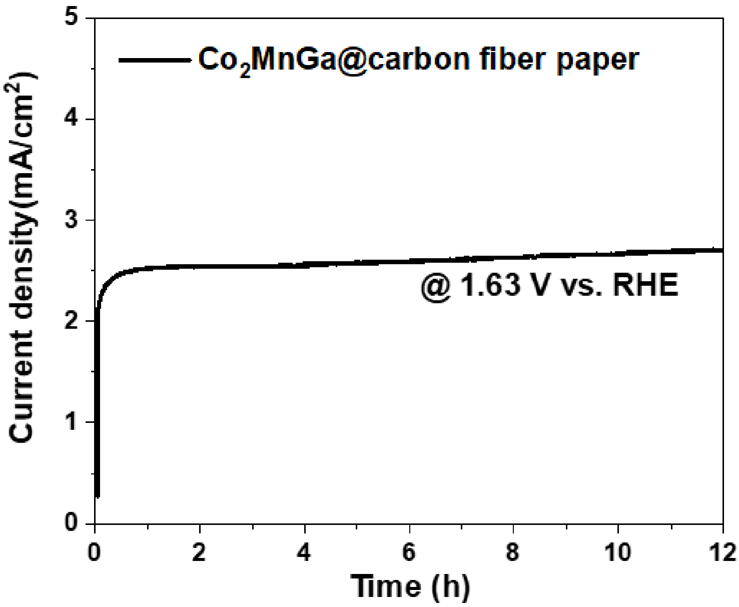

Figure S11. Chronoamperometric curve of an electrode fabricated by depositing Co_2MnGa on carbon fiber paper. A fixed voltage was applied at 1.63 V_{RHE}.

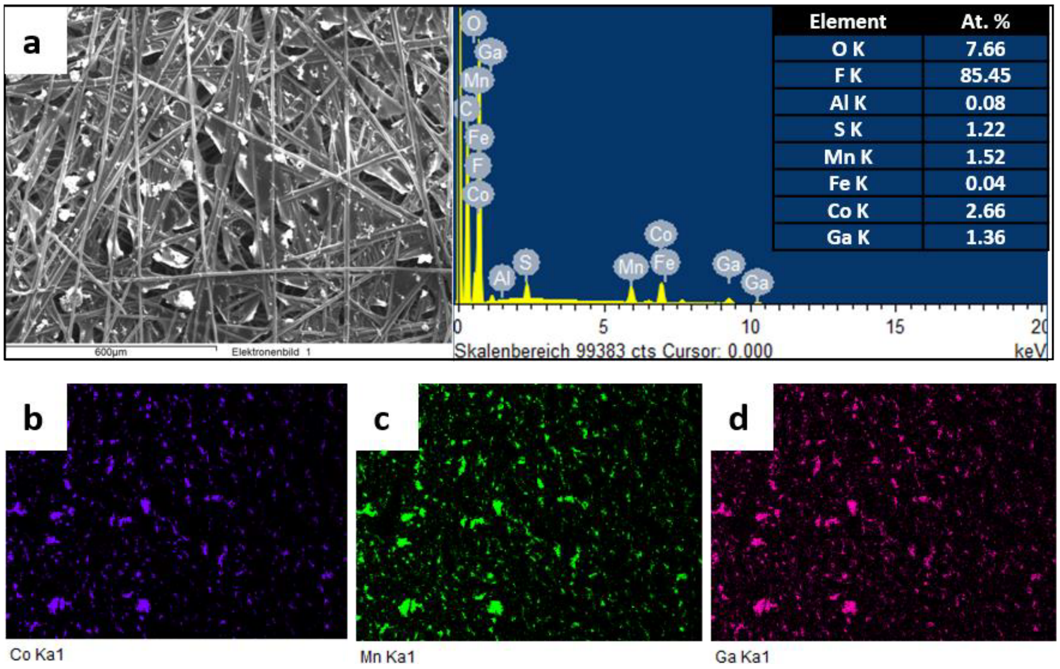

Figure S12. SEM (a) with corresponding EDX analysis, and elemental mapping of Co (b), Mn (c), and Ga (d) of Co_2MnGa on carbon fiber paper before the chronopotentiometry. Note: EDX analysis excluded the content of carbon, and significant amount of F was from Nafion as a binder.

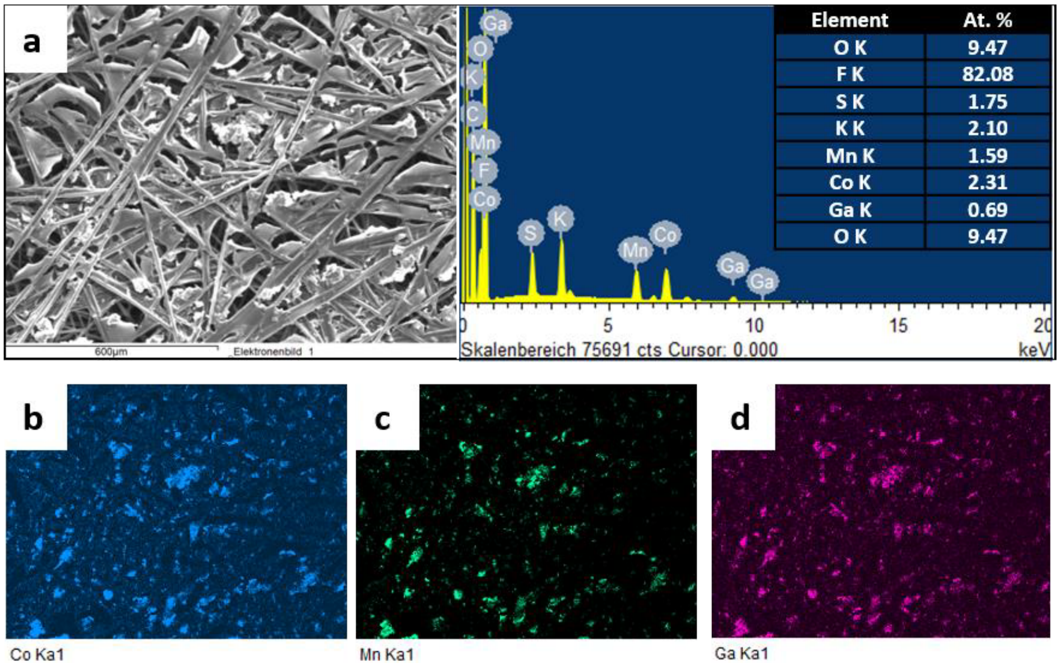

Figure S13. SEM (a) with corresponding EDX analysis, and elemental mapping of Co (b), Mn (c), and Ga (d) of Co_2MnGa on carbon fiber paper after the chronopotentiometry for 12 h. Note: EDX analysis excluded the content of carbon, and significant amount of F was from Nafion as a binder. A small amount of K was due to the residue of KOH on the electrode.

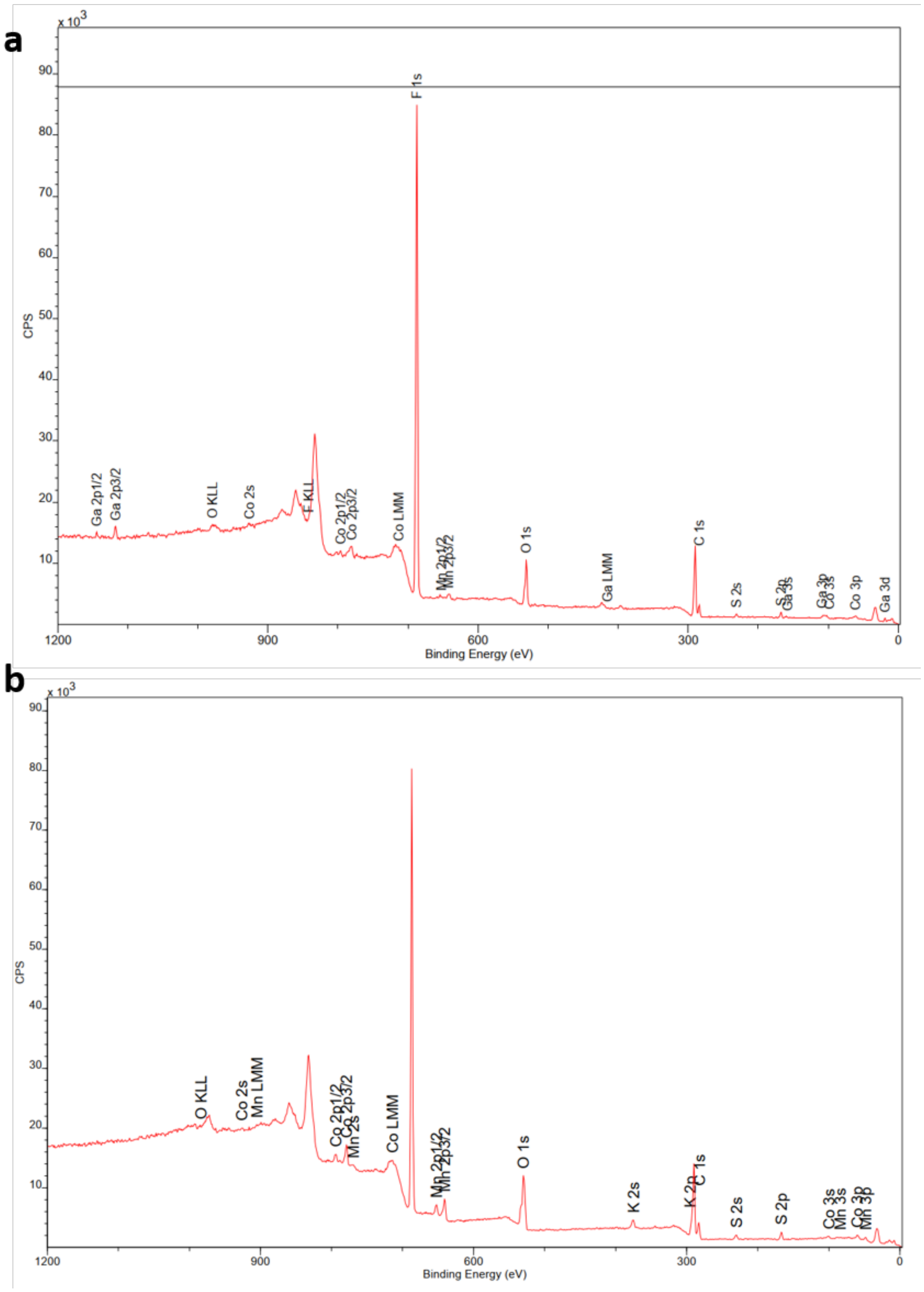

Figure S14. XPS survey of Co_2MnGa@carbon fiber paper before and after OER stability test. C, S, F, and K were detected on the electrode, which were from Nafion and KOH electrolyte.

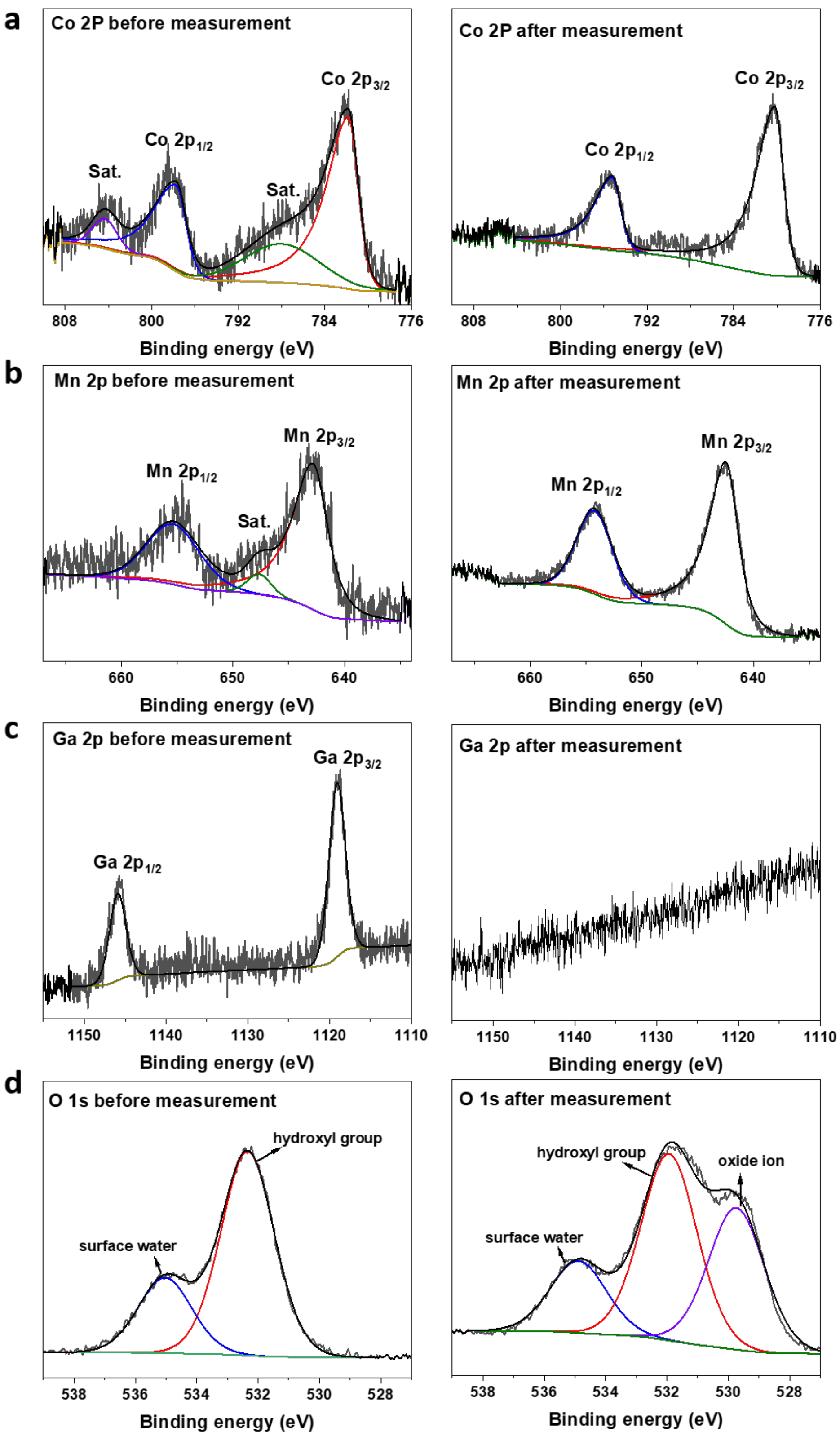

Figure S15. XPS spectra of (a) Co 2p, (b) Mn 2p, (c) Ga 2p, and (d) O 1p region for Co_2MnGa@carbon fiber paper before (left) and after (right) electrochemical test.

High-resolution spectra in Co 2p, Mn 2p, and Ga 2p region show spin-orbit splitting into $2p_{1/2}$ and $2p_{3/2}$ components, as seen in Figure S15a-c. Prior to the electrochemical test, the Co 2p XPS spectrum in Co_2MnGa resembles that of $Co(OH)_2$,[11] suggesting the surface oxidation from Co^{0+} to Co^{2+}. The formation of surface $Co(OH)_2$ could be due to oxidation in contact with air as well as the electrode preparation process where Co_2MnGa was treated in an ultrasound bath. Similarly, surface Mn and Ga exhibited oxidized form compared to the metallic state in pristine Co_2MnGa compound. After conducting electrochemical test, Co $2p_{3/2}$ peak shifted to lower binding (BE) energy in the spectrum of CoO(OH),[11] illustrating surface reconstruction during electrolysis. The formation of surface CoO(OH) has been widely observed for Co-based catalysts in alkaline electrolytes and viewed as the real catalysts during the OER process.[12] On the other hand, no significant change was observed on the Mn 2p spectrum, and Ga was not detected on the surface due to leaching in KOH electrolyte. Furthermore, the O 1s spectra were analyzed to assign the surface oxygen groups (Figure S15d). In O 1s spectrum of fresh Co_2MnGa electrode, the dominant peak at 532.3 eV is assigned to the oxygen in the hydroxyl group,[11] while the peak at higher BE is considered to be molecular water adsorbed on the surface.[13] After electrolysis, an additional peak evolved at a BE of 529.7 eV, corresponding to the oxygen from oxide ions.[11] This suggests the formation of oxyhydroxyl group containing both hydroxyl and oxide ions, in consistency with the change on Co 2p spectra.

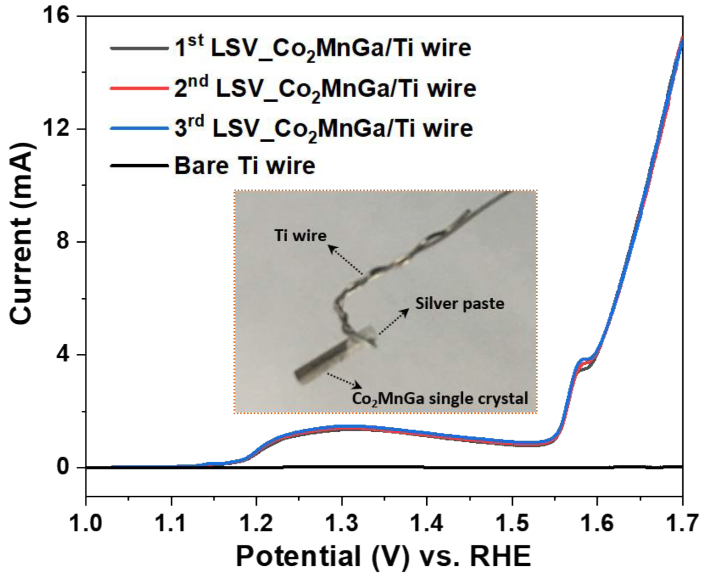

Figure S16. LSV curves of Co_2MnGa/Ti wire electrode and bare Ti wire as electrode. Co_2MnGa single crystal was employed as electrode using silver paste to connect with Ti wire, as seen the structure in the inset photo. The oxidative current was mainly from the surface oxidation on silver paste, with the oxidation peaks centered at 1.23 V_{RHE} and 1.58 V_{RHE} corresponding to the formation of surface Ag_2O and its further oxidation to AgO, respectively.[14]

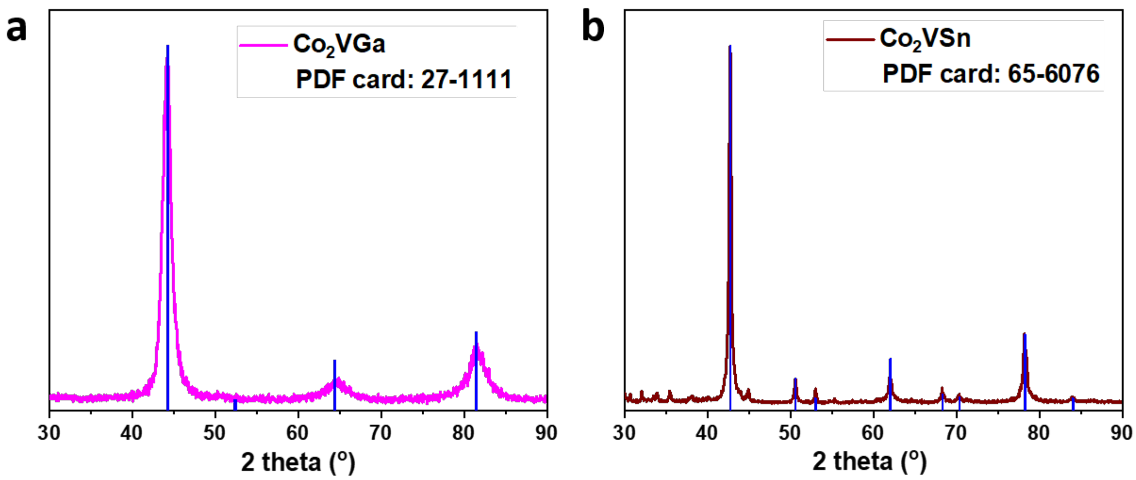

Figure S17. XRD patterns of (a) Co_2VGa and (b) Co_2VSn.

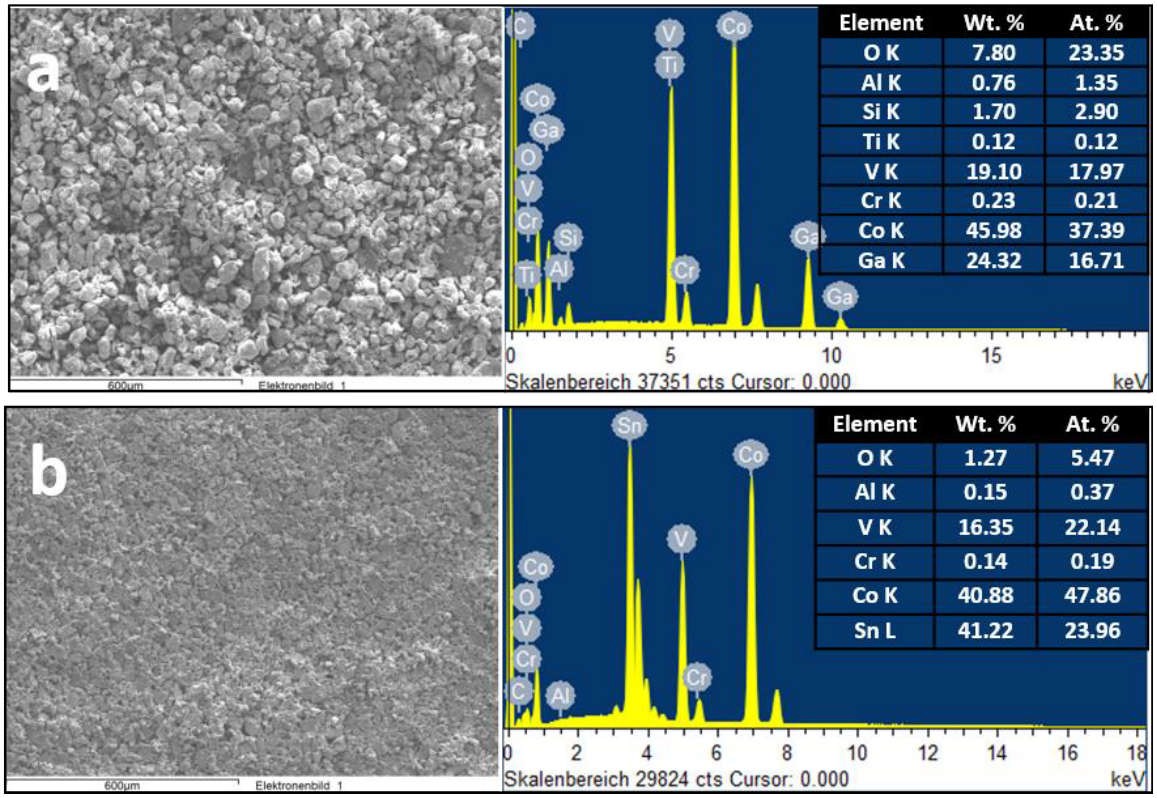

Figure S18. SEM images with corresponding EDX analysis of Heusler compounds: (a) Co_2VGa and (b) Co_2VSn.

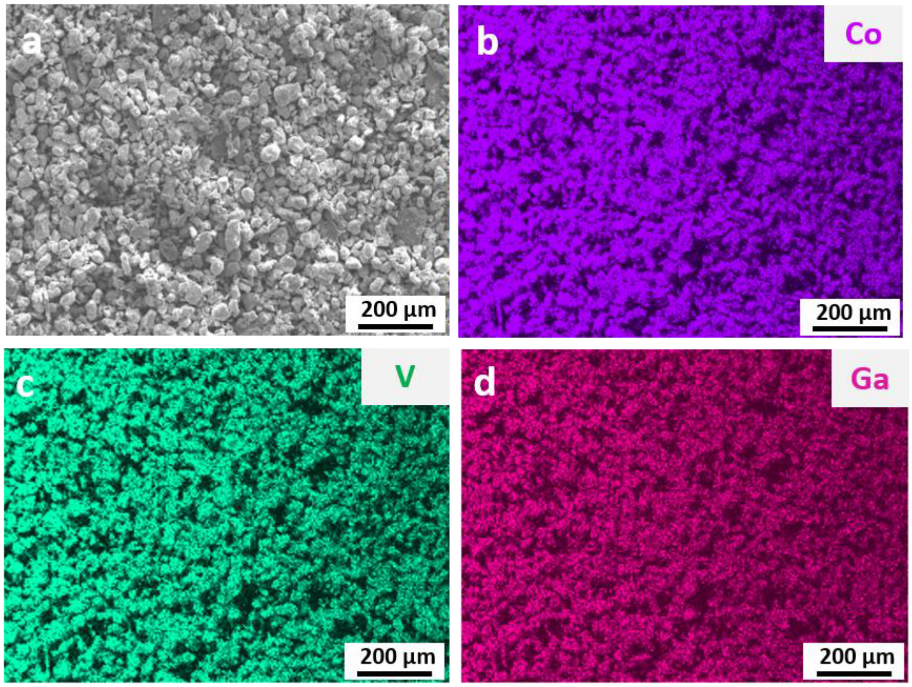

Figure S19. SEM image (a) of Heusler compound Co_2VGa, and corresponding elemental mapping images of (b) Co, (c) V, and (d) Ga.

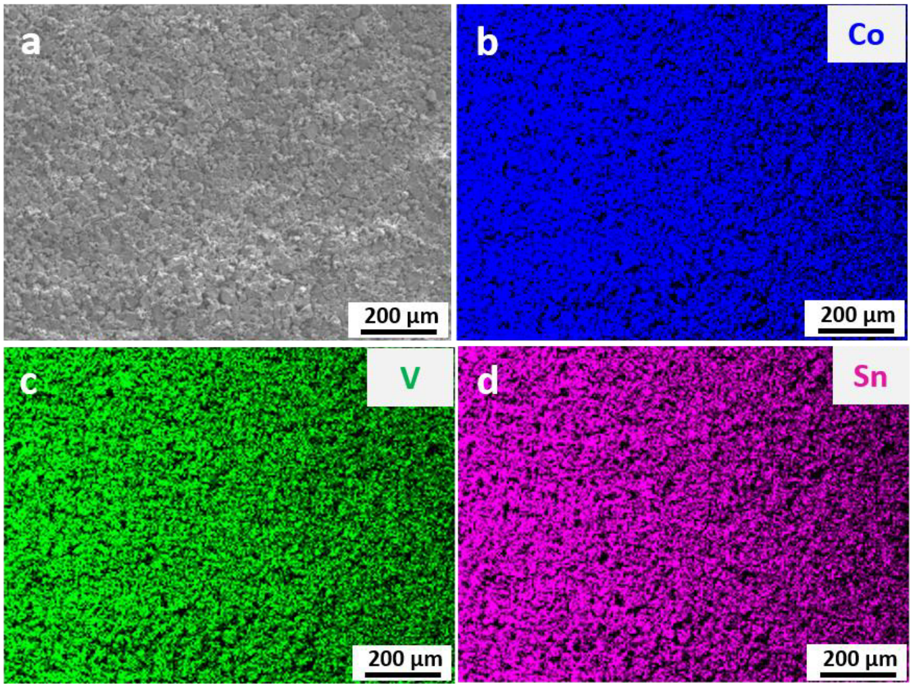

Figure S20. SEM image (a) of Heusler compound Co_2VGa, and corresponding elemental mapping images of (b) Co, (c) V, and (d) Sn.

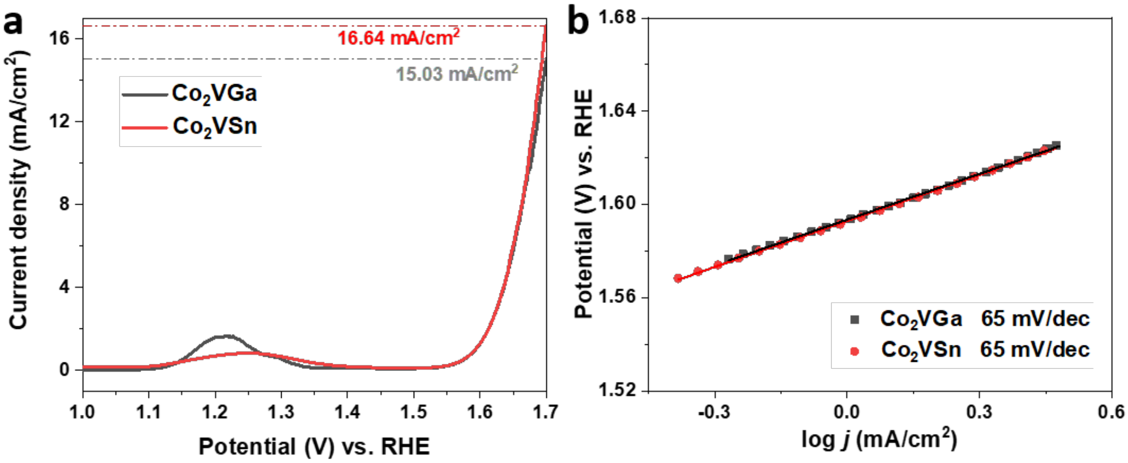

Figure S21. LSV curves (a) and Tafel slopes of Co_2VGa and Co_2VSn.

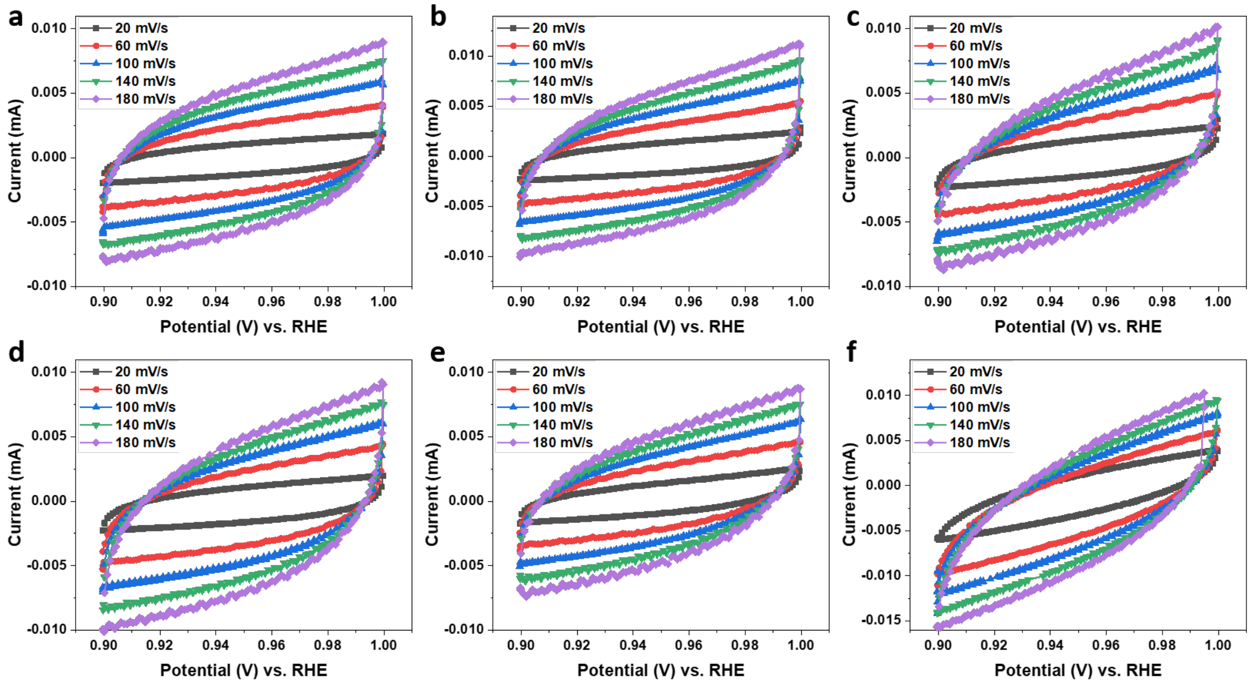

Figure S22. CV curves of Heusler compounds: (a) Co_2MnTi, (b) Co_2MnAl, (c) Co_2MnV, (d) Co_2MnGa, (e) Co_2VSn, and (f) Co_2VGa, which were collected in a non-Faradaic region (1.2 – 1.3 V vs RHE) with different scan rates, 20 mV/s, 60 mV/s, 100 mV/s, 140 mV/s, and 180 mV/s.

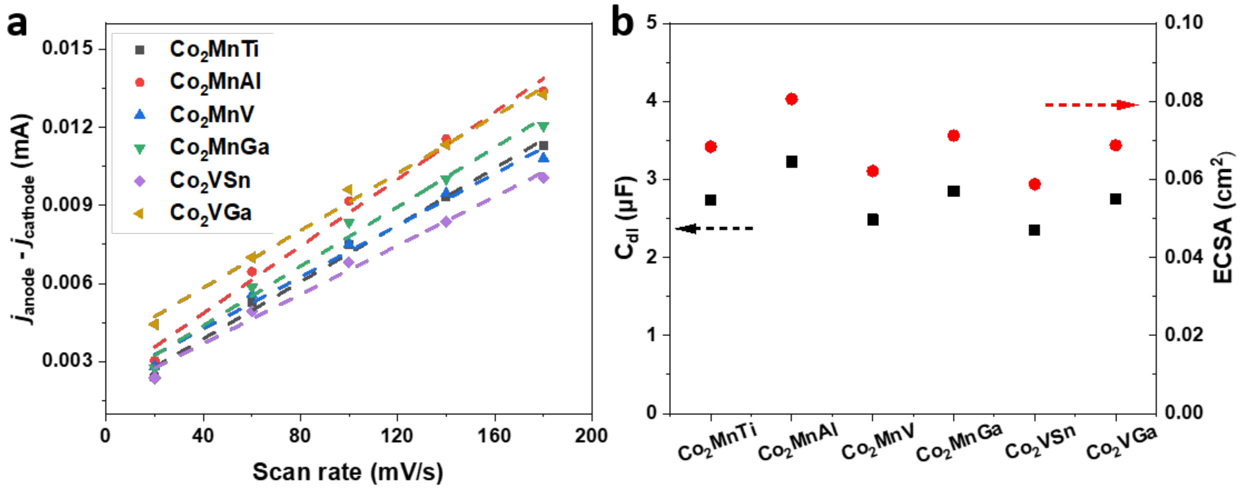

Figure S23. (a) Capacitive current differences (j_{anode} - $j_{cathode}$) at 0.95 V_{RHE} against scan rates, (b) Double-layer capacitance (C_{dl}) and electrochemical surface area (ECSA) of Heusler compounds.

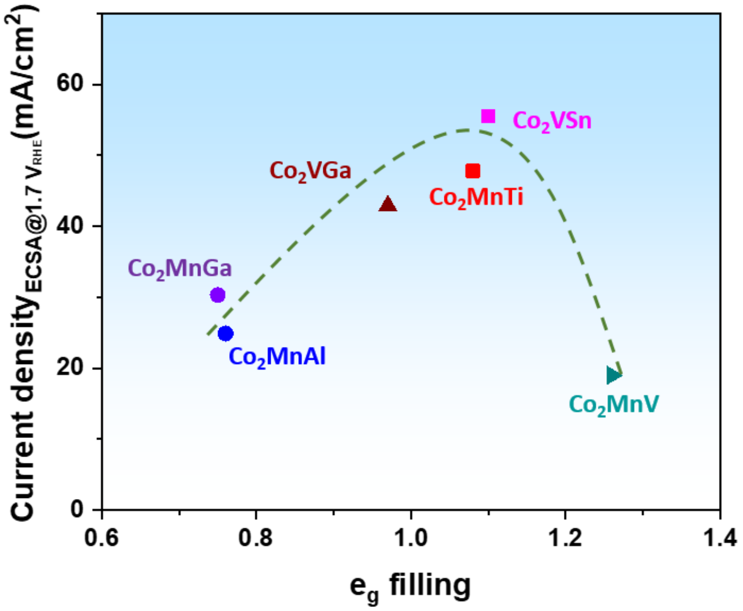

Figure S24. The volcano-shape plot of the OER catalytic activity, defined by the ECSA-normalized current density at 1.7 V_{RHE}, against the occupancy of the e_g electron of Co in Heusler compounds (Co_2MnZ and Co_2VZ). The current density was determined based on the value of ECSA from Figure S23.

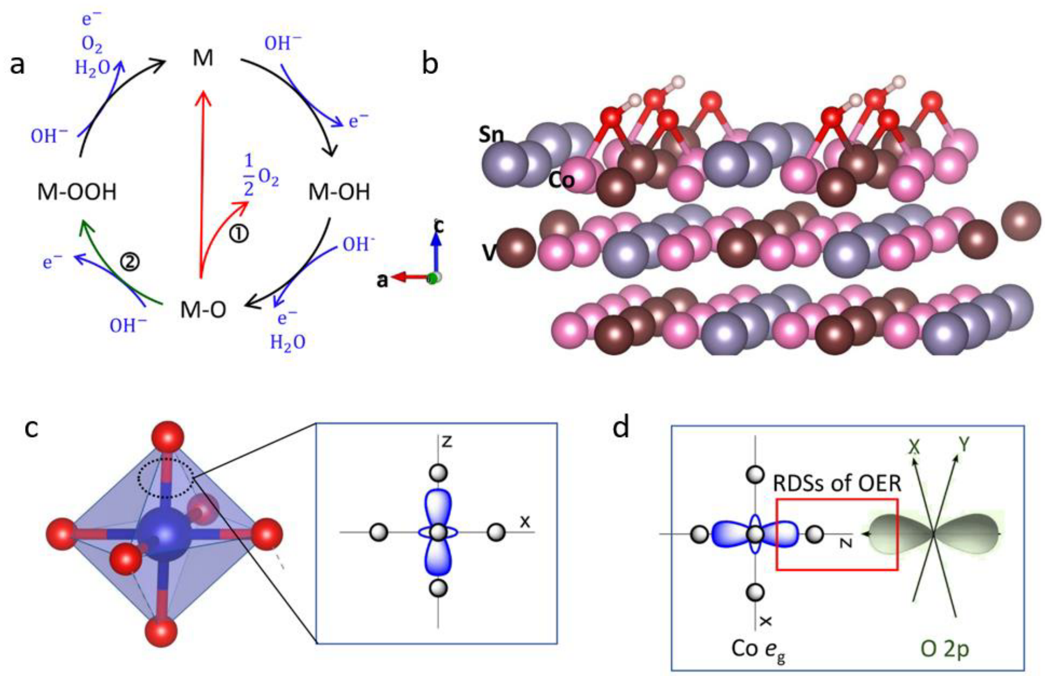

Figure S25. (a) The OER mechanism under alkaline conditions. The red line represents the oxygen formation through the direct reaction of two adjacent *oxo* (M–O) intermediates. The green line indicates that the oxygen generation involves the formation of a peroxide (M–OOH) intermediate, which further couples with OH^- anion with the release of O_2. (b) The adsorption of OOH group at the surface of Co_2SnV (110) surface, which exposes all the three elements at the same time. OOH group is not stable and thus decomposed to OH and O intermediates. (c) The geometry of e_g orbital in the octahedral crystal field. (d) The coupling of the O *2p* orbital with e_g orbital of Co site in Heusler alloy.

Reference:

[1] K. Manna, L. Muechler, T.-H. Kao, R. Stinshoff, Y. Zhang, J. Gooth, N. Kumar, G. Kreiner, K. Koepernik, R. Car, J. Kübler, G. H. Fecher, C. Shekhar, Y. Sun, C. Felser, *Phys. Rev. X* **2018**, *8*, 041045.

[2] C. C. McCrory, S. Jung, J. C. Peters, T. F. Jaramillo, *J. Am. Chem. Soc.* **2013**, *135*, 16977-16987.

[3] G. Kresse, J. Furthmüller, *Comput. Mater. Sci.* **1996**, *6*, 15-50.

[4] G. Kresse, J. Furthmüller, *Phys. Rev. B* **1996**, *54*, 11169-11186.

[5] J. P. Perdew, K. Burke, M. Ernzerhof, *Phys. Rev. Lett.* **1996**, *77*, 3865-3868.

[6] J. P. Perdew, *Phys. Rev. Lett.* **1985**, *55*, 1665-1668.

[7] S. Grimme, J. Antony, S. Ehrlich, H. Krieg, *J. Chem. Phys.* **2010**, *132*, 154104.

[8] H. Han, Y. Dong, L. Fan, K. L. Yao, *J. Appl. Phys.* **2013**, *114*, 143712.

[9] H. C. Kandpal, G. H. Fecher, C. Felser, *J. Phys. D: Appl. Phys.* **2007**, *40*, 1507-1523.

[10] Y. Kurtulus, R. Dronskowski, G. D. Samolyuk, V. P. Antropov, *Phys. Rev. B* **2005**, *71*.

[11] J. Yang, H. W. Liu, W. N. Martens, R. L. Frost, *J. Phys. Chem. C* 2010, 114, 111-119.

[12] A. Bergmann, E. Martinez-Moreno, D. Teschner, P. Chernev, M. Gliech, J. F. de Araujo, T. Reier, H. Dau, P. Strasser, *Nat. Commun.* **2015**, *6*, 8625.

[13] N. A. Merino, B. P. Barbero, P. Eloy, L. E. Cadús, *Appl. Surf. Sci.* **2006**, *253*, 1489-1493.

[14] B. M. Jovic, V. D. Jovic, *J. Serb. Chem. Soc.* **2004**, *69*, 153-166.
